# Non-pharmacological interventions to reduce the symptoms of mild to moderate anxiety in pregnant women. A systematic review and narrative synthesis of women’s views on the acceptability of and satisfaction with interventions

**DOI:** 10.1007/s00737-018-0936-9

**Published:** 2019-01-07

**Authors:** Kerry Evans, Helen Spiby, Jane C. Morrell

**Affiliations:** 1 School of Health Sciences, University of Nottingham, 12th Floor Tower Building, Nottingham, NG7 2RD UK; 2 School of Nursing and Midwifery, University of Queensland, Brisbane, Australia

**Keywords:** Anxiety, Antenatal, Intervention, Pregnancy, Systematic review

## Abstract

To assess women’s views on the acceptability of and satisfaction with non-pharmacological interventions to reduce the symptoms of anxiety in pregnant women. A systematic review and narrative synthesis (Prospero protocol number CRD42015017841). Fourteen included studies were conducted in Australia, Canada, Germany, New Zealand, UK and USA. Interventions were cognitive behavioural therapy, mindfulness, yoga, psychological assessment, supportive and educational based interventions. Studies included women from general antenatal populations and women with anxiety or depression symptoms or risk factors for anxiety or depression. The findings were limited due to the small number of studies evaluating different types of interventions using various study methods. Some studies had too little procedural reporting to allow a full quality assessment. Women’s views on the acceptability of and satisfaction with interventions were overwhelmingly positive. The review highlights women’s motivations for and barriers to participation as well as the benefit women perceived from peer support and individual discussions of their situation. Interventions need to be further evaluated in randomised controlled trials. The inclusion of women’s views and experiences illuminates how and why intervention components contribute to outcomes. Women’s initial concerns about psychological screening and the benefit derived from peer support and individual discussion should be noted by providers of maternity care.

## Introduction

The Diagnostic and Statistical Manual of Mental Disorders (DSM-V) (American Psychiatric Association 2013) described the symptoms for the most prevalent anxiety disorders: generalised anxiety disorder (GAD), panic disorder, agoraphobia, obsessive compulsive disorder, specific phobias and social anxiety disorder. Although specific anxiety disorders have specific symptoms, they share common symptoms which include excessive and intrusive worrying, feeling overwhelmed, angry or scared, irritability, fatigue, difficulty concentrating and sleeping, an elevated sensitivity to threat and a bias to interpret ambiguous information in a negative way (Craske et al. 2009; National Institute for Health and Care Excellence, NICE 2011; Highet et al. 2014; Staneva et al. 2015). In pregnancy, concerns over the wellbeing of the baby, the labour and birth or parenting may present as predominant features (Staneva et al. 2015; Vythilingum 2009). Pregnant women with anxiety have reported feeling a loss of control over their bodies and feeling confused by ambiguous information about pregnancy and labour (Highet et al. 2014; Keeton et al. 2008; Staneva et al. 2015). Women with a previous or existing mental illness, those who have poor partner or social support, women who are socially isolated, women from a low socio-economic background, those who are exposed to violence or abuse, women who are substance misusers, women with unplanned or unwanted pregnancies or those who have had a previous negative experience of pregnancy or birth are especially vulnerable to developing symptoms of anxiety in pregnancy (Biaggi et al. 2016; Staneva et al. 2015).

Reported prevalence of anxiety disorders in pregnancy varies from 10 to 16% (Goodman et al. 2014; National Institute for Health and Care Excellence (NICE) 2018; Rubertsson et al. 2014) and has been reported as 15–16% in UK and Canadian community samples (Heron et al. 2004; Fairbrother et al. 2016). Elevated and prolonged anxiety in pregnancy has been associated with pre-term birth, fetal growth restriction (Ding et al. 2014; Littleton et al. 2007; Rich-Edwards and Grizzard 2005) and childhood behavioural problems (Blair et al. 2011; Cardwell 2013; Davis and Sandman 2010; Glover 2014; Stein et al. 2014). Mild to moderate psychological distress can be debilitating and have a negative effect on women’s general functioning (Furber et al. 2009). It is associated with post-traumatic stress disorder (Czarnocka and Slade 2000; Iles et al. 2011) and postnatal depression (Heron et al. 2004; Coelho et al. 2011).

### Rationale

Women with severe anxiety symptoms require assessment and management from specialist mental health services. In the management of women with mild to moderate mental health problems, the aim is to prevent an escalation of symptoms and improve a woman’s quality of life (NICE 2018). All women identified with mild to moderate mental health problems should have access to a range of support such as wellbeing advice, guided self help, motivational interviewing, cognitive behavioural therapy (CBT) and medication (Department of Health (DOH) 2012). However, services to support the emotional wellbeing of women need to be strengthened in order to provide suitable and timely support and treatment to help avoid illness (Maternal Mental Health Alliance 2013). The NICE guideline for perinatal mental health (NICE 2018) suggested that non-pharmacological interventions such as low intensity psychological interventions may benefit women with symptoms of mild to moderate anxiety. Social support, assisted self-help and CBT are proposed in The Healthy Child Programme (DOH 2009) as possible interventions to support pregnant women with anxiety. However, evidence of the effectiveness of such interventions has not yet been established.

### Objectives

The review aimed to answer the following questions:

- How acceptable for pregnant women are non-pharmacological interventions for reducing the symptoms of mild to moderate anxiety?
- How beneficial do pregnant women consider non-pharmacological interventions to be in reducing the symptoms of mild to moderate anxiety in pregnancy?

## Methods

### Protocol and registration

A systematic review was conducted following the Centre for Reviews and Dissemination guidelines (CRD 2009). The narrative synthesis followed the guidelines by Popay et al. (2006). The review protocol was registered on the PROSPERO database at the CRD (Evans et al. 2015 CRD42015017841).

### Eligibility criteria

#### Participants

Pregnant women of all parities across the three trimesters of pregnancy. Women less than 18 years of age and women who lacked capacity to provide informed consent were excluded from the study. In addition, pregnant women with complex social factors were not included (pregnant women who misuse alcohol and/or drugs; are recent migrants, asylum seekers or refugees; have difficulty reading or speaking English; experience domestic abuse) (NICE 2010). Women under the care of specialist mental health services or women with severe symptoms of anxiety were excluded. Studies used various measurement techniques to assess eligibility. Some studies used anxiety scales with dimensional cut-off scores for mild, moderate and severe anxiety to assess eligibility (Brunton et al. 2015). Studies using dimensional anxiety scales who included women with severe scores were excluded (Table 2).

#### Interventions

Non-pharmacological interventions were classified as (1) psychological, (2) mind-body, (3) educational and (4) supportive interventions.

#### Outcomes

The primary outcome was women’s views on the acceptability of and satisfaction with interventions.

#### Study design

Quantitative or qualitative studies which assessed women’s views on the acceptability of and satisfaction with an intervention.

### Information sources

A systematic search of the following electronic databases was undertaken in January 2015 and updated in June 2018:

Medline (Medical Literature Analysis and Retrieval System Online), CINAHL (Cumulative Index to Nursing and Allied Health Literature), Maternity and Infant Care database from MIDIRS (Midwives Information and Resource Service), PsycINFO, The Cochrane Library, EMBASE (Excerpta Medica Database), CRD (Centre for Reviews and Dissemination), SSCI (Social Sciences Citation Index), ASSIA (Applied Social Sciences Index and Abstracts), HTA (Health Technology Assessment) Library, JBI (Joanna Briggs Institute) Evidence-Based Practice Database and AMED (The Allied and Complementary Medicine Database). Visually scanned reference lists from relevant primary studies and reviews identified two additional studies for inclusion.

### Search

The search was limited to studies conducted in countries with similar maternity care to the UK and published in English since 1990. This period reflects the time that non-pharmacological interventions have been recommended to support women’s mental health during pregnancy (DOH 1999). Search terms included pregnancy, antenatal, anxiety, intervention, trial, review, women’s views, acceptability and satisfaction. A full search strategy is included in Appendix 1.

### Study selection

Potentially eligible papers were retrieved for full text assessment which was conducted independently by two researchers. Any disagreements were resolved by a third researcher.

### Data collection process

A pre-piloted data extraction form was completed independently by two researchers for each included study.

### Quality assessment

The Critical Appraisal Skills Programme (CASP 2014) for assessing the methodological quality of qualitative studies and the Critical Appraisal Checklist for a Questionnaire Study (Boynton and Greenhalgh 2004) were used to assess the quality of studies included in the review.

### Analysis strategy

Data analysis and synthesis followed the suggested frameworks for conducting a narrative synthesis (Popay et al. 2006). Qualitative and quantitative studies which addressed the research questions were used to explore similarities and/or differences in the common themes (Popay et al. 2006). Each study was first described with reference to the context as intended by the original research (Jensen and Allen 1996). Secondly, a table of key concepts was produced to explore the homogeneity of themes, noting any discordance. Themes emerged from the similarities and contradictions between the study findings (Walsh and Downe 2005). The next phase involved translating the study findings using concepts that could be applied to all or some of the studies.

### CERQual assessment

The Confidence in the Evidence from Reviews of Qualitative Research (CERQual) approach was used to assess the extent to which the review findings from the qualitative studies represented the phenomenon of interest (Lewin et al. 2015; The Cochrane Collaboration 2011). The process required an individual assessment of the studies which contributed to a review finding. Assessment components included methodological limitations, relevance to the review questions, adequacy of data and coherence (whether the finding was well grounded in data with a convincing explanation). After assessing each of the four components, an assessment of the overall confidence in each review finding was made. Each review finding was assessed as having a high, moderate, low or very low confidence rating (Lewin et al. 2015).

## Results

### Study selection

**Fig. 1:**
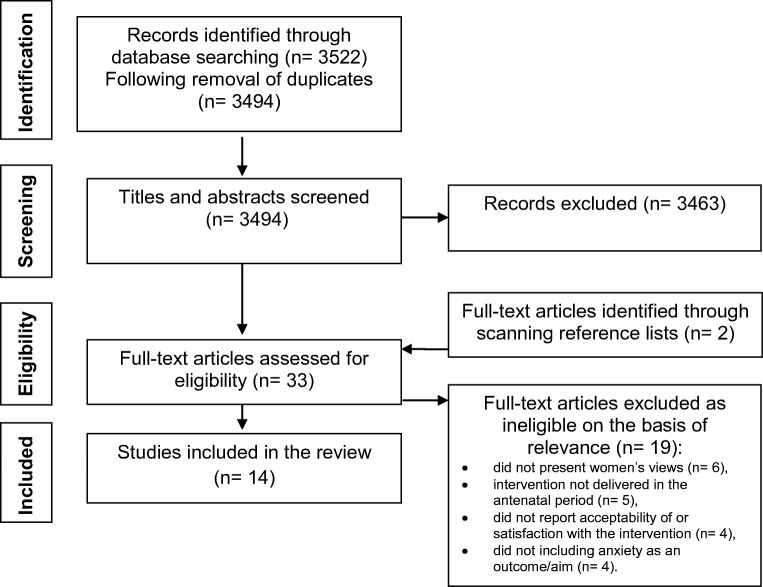
PRISMA flow diagram: women’s views of interventions

The search identified 3522 potentially eligible papers which were assessed on the information provided in the abstract using the review eligibility criteria. Duplicate papers were removed. Potentially eligible papers (*n* = 3494) were retrieved for full text assessment. Excluded papers (*n* = 3643) (1) did not report interventions delivered in pregnancy, include women’s views or report non-pharmacological interventions; (2) included women with severe mental health concerns or complex social factors. The literature search and inclusion process is detailed in the PRISMA Flow diagram (Moher et al. 2009) (Fig. 1).

### Study characteristics

The 14 included studies, conducted in Australia, Canada, Germany, New Zealand, UK and USA, were reported from 2009 to 2015. Components of the interventions are detailed in Table 1. There were 800 women in the included studies. Sample sizes ranged from four women (Breustedt and Puckering 2013) to 298 women (Brugha et al. 2015). Overall, from the 800 participants, 204 women provided views about the interventions via questionnaires or qualitative interviews.

**Table 1:** Data extraction from the studies included in the review

*First author Country* *Year*	*Intervention category (duration)*	*Primary outcome* *(secondary outcome)*	*Gestation at start/post intervention (weeks of pregnancy)*	Study type *Description of intervention **Facilitator/facilitator training	*Method and timing of outcome measure: acceptability/ satisfaction/ beneficence (n=)*	*Quality assessment of the methods used to investigate the acceptability/satisfaction/beneficence of the intervention*
McGregor Canada 2014	Psychological (6 individual sessions: 8 weeks)	1. Depression 2. Anxiety 3. Healthcare/ medication utilisation (Intervention evaluation)	20*/*28	Pilot quasi-experimental trial * 10 min CBT sessions: education and behavioural activation; cognitive restructuring; inter-connectedness of thoughts, feelings and behaviours. ** Physicians*/*two hour training session provided by a psychologist.	Questionnaire Six weeks post-partum (*n* = 19)	No information provided on the development or the validity*/*reliability of the questionnaire. Questionnaires contained brief open and closed questions to assess women’s experiences and satisfaction with the CBT intervention. The authors reported that content analysis was conducted on the open ended questions, no further information provided.
Milgrom Australia 2015	Psychological (8 individual sessions: 8 weeks)	1. Depression 2. Anxiety (Infant outcomes, satisfaction)	20 (mean) / 29 (approx)	Pilot RCT * CBT sessions: ‘Beating the Blues Before Birth’ (Lewinsohn et al. 1984): relaxation; cognitive strategies; support networks; partner sessions; parenting skills; relationship issues and anxiety. ** Psychologists*/*Trained in pregnancy-specific CBT.	Questionnaire Post-intervention approx 29 weeks (*n* = 19)	No information provided on the development or the validity*/*reliability of the questionnaire. Questionnaire contained six items on the helpfulness of and satisfaction with the intervention (Likert scale). Results presented as simple descriptive statistics.
Bittner Germany 2014	Psychological (8 group sessions: 8 weeks)	1. Depression 2. Anxiety (Fear of childbirth, social support, intervention evaluation)	16 (mean) / 24	RCT * CBT sessions: coping strategies; self-assurance; problem solving; discussions about anxiety; prevention; treatment; future challenges. ** Psychologist*/*CBT Training and supervision.	Questionnaire Post-intervention – 24 weeks (*n* = 36)	No information provided on the validity*/*reliability of the questionnaire. Questionnaire contained items about participants’ experience of and satisfaction with the intervention (Likert scale). The RCT had a high rate of attrition (46%). Results presented as simple descriptive statics.
Thomas Australia 2014	Psychological*/*Educational (6 group sessions: 12 weeks)	1. Depression 2. Anxiety 3. Maternal attachment (acceptability, satisfaction)	26 (mean) */*NR	Pilot study * Behavioural self-care; psycho-education; IPT (social support, communication, role transitions, mental health warning signs); parent-infant relationship. ** Clinical psychologist and parent-infant mental health clinicians*/*experienced in CBT and IPT.	Questionnaire Post-intervention – third trimester (*n* = 30)	The authors used a validated questionnaire, the CSQ-8 to assess satisfaction. There was no information on the development of the intervention feedback forms. Results were presented as simple descriptive statics.
Brugha UK 2015	Psychological*/*Supportive (up to 3 individual sessions: 22 weeks)	1.Depression (Anxiety and satisfaction)	22 / 34 (approx)	Pilot cluster RCT * Care from midwives with additional training on: assessment of depressive symptoms; CBA; facilitating and maintaining therapeutic relationships; Five Areas approach (Williams et al. 2008) ** Midwives*/*Based on training by Morrell et al. (2009) and adapted for pregnancy.	Qualitative interviews Post-intervention – approx 34 weeks (*n* = 8)	A stratified subsample of intervention group women with EPDS scores of 12 or more and less than 12 were invited to take part in a qualitative evaluation of the pilot. Limited reporting of the methods of data collection. The authors described the data analysis method and provided quotations to support the findings.
Breustedt Scotland, UK 2015	Psychological*/*Social support (8 group sessions)	1. Participants’ experience of the intervention	NR	Qualitative study * ‘Mellow Bumps’ psychological and practical techniques to reduce anxiety and promote wellbeing in vulnerable pregnant women; encouraged women to make social connections, share information; addressed individual concerns.	Qualitative interviews Post-partum period (*n* = 4)	Women who had completed the intervention and maintained contact participated. Authors state this may be related to positive experiences and non-attendees may hold different views. Authors included a description of the topic guide, data analysis method and participant quotations. A second researcher assessed for possible bias in the analysis process.
Côté-Arsenault US 2014	Supportive (approx 5 individual sessions: 20 weeks)	1. Anxiety 2. Depression (Intervention evaluation)	14 (mean) */*NR	RCT * Supportive care for women pregnant after perinatal loss: pregnancy diary, information, skills to reduce anxiety and depression; prenatal attachment. Based on the caring process (Swanson, 1993). ** Nurses with additional training*/*NR	Qualitative Interviews Six to nineteen months post-partum (*n* = 12)	Qualitative interviews conducted with the intervention group participants. Limited reporting of the methods of data collection. The authors described the data analysis method and provided participant quotations to support the findings. Used member checking in the data analysis procedure.
*First author Country* *Year*	*Intervention category (duration)*	*Primary outcome* *(secondary outcome)*	*Gestation at start/post intervention (weeks of pregnancy)*	*Study type* *Description of intervention **Facilitator/facilitator training	*Method and timing of outcome measure: acceptability/ satisfaction/ beneficence (n=)*	*Quality assessment of the methods used to investigate the acceptability/satisfaction/beneficence of the intervention*
Beddoe US 2009	Mind body (7 group sessions)	1. Stress 2. Anxiety 3. Pain 4. Cortisol levels 5. Acceptability	13–32 / NR	Feasibility study * Mindful yoga intervention combined elements of the Iyengar yoga, MBSR, relaxation and stress management. ** Yoga MBSR instructor*/*experienced Iyengar yoga instructor with extensive training in MBSR.	Questionnaire Post-intervention (*n* = 16)	The authors reported that the findings were limited by the inclusion of a small self-selected sample of women. No information provided on the validity*/*reliability of the questionnaire. Participants rated the acceptability of and satisfaction with the intervention. Results presented as simple descriptive statics.
Cornsweet Barber New Zealand 2013	Mind body (Individual self-help material)	1. Acceptability of the intervention and usability of the self-help material	Second and third trimesters of pregnancy*/*NR	Feasibility study * computerised self-help package using bio-feedback to teach relaxation and mindfulness skills ** self-help	Qualitative Interviews Post-intervention - Second and third trimester (*n* = 9)	The authors reported the findings were limited by the inclusion of a small self-selected sample of pregnant women. Limited reporting of the methods of data collection and qualitative data analysis. The authors presented a small number of examples of participant quotations to support the findings
Davis US 2015	Mind body (8 group sessions: 8 weeks)	1. Depression 2. Anxiety 3. Positive and negative affect (satisfaction, adherence)	21 (mean) / 28–29	RCT * Ashtanga Vinyasa yoga modified for pregnancy. Instructional video for home use. ** Yoga instructor*/*Experience in prenatal yoga	Questionnaire Post-intervention (*n* = 23)	The questionnaire was completed by women in the intervention group. The authors used validated questionnaires, the CSQ-8 to assess satisfaction and a credibility scale questionnaire. The results were presented as simple descriptive statics.
Dunn Australia 2012	Mind body (8 group sessions: 8 weeks)	1. Depression 2. Anxiety 3. Stress 4. Self-compassion 5. Mindfulness Awareness (Participants’ experience)	12–28 / NR	Pilot quasi-experimental study * Based on MBCT programme (Segal et al. 2002): awareness of each moment; cognitive model; taking a wider perspective; fostering an attitude of acceptance; relating to negative thoughts; managing warning signs. ** Psychiatrist, counsellor*/*accredited MBCT facilitators.	Qualitative Interviews Six weeks post-partum (*n* = 10)	Qualitative interviews conducted with the intervention group participants. The authors employed a non-randomised design and reported that the intervention and control groups were unbalanced at baseline (history of anxiety/depression). Limited reporting of the methods of data collection and data analysis. Presented extensive examples of participant quotations.
Goodman US 2014	Mind body (8 group sessions: 8 weeks)	1. Anxiety 2. Depression 3. Self-compassion 4. Mindfulness (Intervention evaluation)	6–27 / NR	Pilot study * Stress management: using imagination to induce feelings of comfort. Based on hypnotherapeutic methods. ** Stress management expert*/*NR	Questionnaire Post-intervention – second and third trimester (*n* = 23)	Open ended questions were used to elicit qualitative feedback concerning participation in the intervention. Qualitative content analysis was used to analyse the data with little further information provided. Quotations were presented to support the findings.
Woolhouse Australia 2014	Mind body (6 group sessions: 6 weeks)	1. Stress 2. Depression 3. Anxiety (Participants’ experience)	11–34 / 17–40	Pilot RCT * ‘MindBabyBody’: breathing practice; body-scan; mindfulness of pain and thoughts; meditation; self-compassion; mindfulness skills in motherhood. ** Psychologist and Psychiatrist*/*Training in facilitation of mindfulness groups.	Qualitative Interviews Post-intervention – 17-40 weeks (*n* = 4)	Qualitative interviews with a small self selected sample of intervention group participants. Limited reporting of the data collection procedures. A detailed description Interpretative Phenomenological Analysis (IPA) procedure was reported and quotations were provided to support the findings.
Darwin UK 2013	Other (individual psycho-social assessment)	1. Consider how perinatal psychosocial assessment may act as an intervention	18 (mean) / 25	Mixed methods study * Participated in a psychosocial assessment at the pregnancy booking appointment as part of routine clinical practice ** Midwives and Healthcare Professionals	Qualitative Interviews Time 1: 10–12 weeks Time 2: 28–36 weeks Time 3: 7–13 weeks post-partum (*n* = 22)	Author employed sequential mixed methods sampling (cases where the most could be learnt in relation to the research questions). Women participated in up to 3 qualitative interviews. Field notes and a reflective dairy were used to assist analysis. Presented a clear and transparent approach to the data collection process. Participant quotations presented to support the findings. A second researcher completed data analysis to reduce bias. The author described the use of prolonged engagement, member checking and searching for alternative explanations in the analysis procedure.

*RCT* randomised controlled trial, *IPT* inter-personal therapy, *CBA* cognitive behavioural approach, *CBT* cognitive behavioural therapy, *MBSR* mindfulness-based stress reduction, *MBCT* mindfulness-based cognitive therapy, *PND* postnatal depression, *NR* not reported

### Participants

In four studies, women were recruited from a general pregnant population. In eight studies, pregnant women with a history of mood concerns or elevated anxiety or depression scores were recruited (Table 2). Two studies included women with social risk factors and pregnant women with a history of previous pregnancy loss. Many of the studies used one of the self-report measures listed in Table 3 for participant inclusion.

**Table 2:** Psychological characteristics of participants in the included studies

Intervention type	First author*/*year	Mean anxiety score at baseline	Exclusions based on mental health history, diagnosis or treatment
*Interventions for women with elevated anxiety scores or risk factors*			
Mind Body Stress management Group sessions	Goodman 2014	BAI 12	DSM criteria: bipolar disorder, substance dependence disorder, psychotic disorder, anxiety disorder other than GAD that was more severe than GAD symptoms or diagnosis; initiated or increased dose of pharmacological treatment for depression*/*anxiety within past 6 weeks; participating in psychotherapy > 2 times per month; received CBT or stress reduction program in the past 12 months.
*Interventions for women with elevated anxiety and/or depression scores or risk factors*			
Psychological CBT Group sessions	Bittner 2014	STAI-S 38	Severe anxiety, depression, bipolar or schizophrenic disorder or lithium/anti-psychotic drug intake
Psychological CBT Individual sessions	Milgrom 2015	BAI 19	Major health problems, major psychiatric disorders for which the treatment was not designed (psychotic and bipolar disorders; not exclude anxiety disorders), current use of other psychological programmes,
Psychological Educational, CBT, IPT Group sessions	Thomas 2014	STAI-S 53	Currently using illicit drugs or excessive amounts of alcohol, current psychotic symptoms, or acute risk of suicide.
Mind Body Yoga	Davis 2015	STAI-S 39	(1) lifetime diagnosis of schizophrenia or schizoaffective disorder, bipolar disorder, current psychosis, organic mental disorder or pervasive developmental delay, or any other disorders that necessitated priority treatment not provided by the study protocol, (2) imminent suicide or homicide risk (3) high risk pregnancy
Mind Body Mindfulness Group sessions	Woolhouse 2014	STAI-S 36	Current substance abuse; severe suicidal ideation
Psychological assessment	Darwin 2013	NR	NR
Psychological*/*Supportive	Breustedt 2015	NR	NR
*Interventions for women with elevated depression scores or risk factors*			
Psychological CBT Individual sessions	McGregor 2014	STAI-S 45	Use of antidepressant or antipsychotic medication
*Interventions for women with a history of pregnancy loss*			
Supportive care	Côté-Arsenault 2014	NR	Uncontrolled medical or mental illness
*Interventions for a general population of pregnant women*			
Psychological, Supportive CBA	Brugha 2015	NR	In receipt of treatment from specialist mental health services
Mind Body Mindful Yoga Group sessions	Beddoe 2009	STAI- 26.7*/*30.4	Current psychiatric illness; currently used medications for pain, sleep, depression, or anxiety.
Mind Body Mindfulness relaxation Self-help material	Cornsweet Barber 2013	NR	NR
Mind Body MBCT Group sessions	Dunn 2012	NR	Current psychosis or active substance abuse

**Table 3:** Anxiety self-report measures used in the included studies

BAI	Beck Anxiety Inventory (Beck et al. 1988)
BDI	Beck Depression Inventory (Beck et al. 1988)
EPDS	Edinburgh Postnatal Depression Scale (Cox et al. 1987)
GAD-2	Generalised Anxiety Disorder–2 items (Spitzer et al. 2006)
GAD-7	Generalised Anxiety Disorder–7 items (Spitzer et al. 2006)
PDQ	Prenatal Distress Questionnaire (Yali and Lobel 1999)
PHQ-9	Patient Health Questionnaire–9 (Kroenke et al. 2001)
PSWQ	Penn State Worry Questionnaire (Meyer et al. 1990)
STAI	State-Trait Anxiety Index (Spielberg et al. 1970)

Women were mainly recruited into studies while attending antenatal appointments in hospital and community locations. Women either self-selected into studies or were referred by healthcare professionals (HCP).

*RCT* randomised controlled trial, *IPT* inter-personal therapy, *CBA* cognitive behavioural approach, *CBT* cognitive behavioural therapy, *MBSR* mindfulness-based stress reduction, *MBCT* mindfulness-based cognitive therapy, *PND* postnatal depression, *NR* not reported

### Interventions

Five studies evaluated psychological interventions: Cognitive behavioural therapy (CBT) (Bittner et al. 2014; Mcgregor et al. 2013; Milgrom et al. 2015); cognitive behavioural approach (CBA) (Brugha et al. 2015); psycho-educational and inter-personal therapy (IPT) (Thomas et al. 2014); and psychological, practical techniques and social support to promote wellbeing (Breustedt and Puckering 2013).

Six studies evaluated mind body interventions: hypnotherapeutic techniques and stress management (Goodman et al. 2014), mindfulness-based cognitive therapy (MBCT) (Dunn et al. 2012), mindfulness (Woolhouse et al. 2014), mindful yoga and mindfulness-based stress reduction (Beddoe et al. 2009) and yoga (Davis et al. 2015).

One study evaluated a supportive intervention: home visits by nurses (Côté-Arsenault et al. 2014) and one study considered how perinatal psychosocial assessment may act as an intervention (Darwin et al. 2013). Many of the psychological and supportive interventions also included components of parent education, relaxation and/or social support.

### Outcomes

The included studies reported women’s views and responses to questions about the level of satisfaction, perceived benefits, acceptability, and relevance of interventions.

### Study type

Qualitative and quantitative studies were included. Four studies conducted interviews with pregnant women (Brugha et al. 2015; Cornsweet Barber et al. 2013; Darwin et al. 2013; Woolhouse et al. 2014). Three studies interviewed postnatal women about their participation during pregnancy (Breustedt and Puckering 2013; Côté-Arsenault et al. 2014; Dunn et al. 2012). Goodman et al. (2014) collected qualitative data during pregnancy from a post-intervention questionnaire.

There were six cross-sectional surveys, administered post-intervention during the second and/or third trimesters of pregnancy (Beddoe et al. 2009; Bittner et al. 2014; Davis et al. 2015; Mcgregor et al. 2013; Milgrom et al. 2015; Thomas et al. 2014). The quality assessment of the included studies is presented in Table 2.

### Quality appraisal

A summary of the quality assessment of the included studies is presented in Table 1. Many surveys had limited reporting of the questionnaire design, validity and reliability, administration and analysis all included a questionnaire as part of a larger quantitative evaluation. Results were presented as numbers and percentages with individual questionnaire item scores and brief descriptive statements of agreement or disagreement from participants.

### GRADE-CERQual assessment

The CERQual components were used to assess the overall confidence in the findings of the seven qualitative studies:

- Two studies were assessed as having moderate methodological limitations (Cornsweet Barber et al. 2013; Dunn et al. 2012). Five studies were assessed as low for methodological limitations.
- One study was assessed as having moderate coherence (findings well-grounded in the data) (Cornsweet Barber et al. 2013). Six studies were assessed as being highly coherent.
- Two studies were assessed as being moderately relevant to the context of the review questions (Côté-Arsenault et al. 2014; Darwin et al. 2013). Five studies were assessed as being highly relevant.
- The adequacy of data was assessed as being highly adequate in six studies where the authors provided detailed accounts of women’s views and experiences and used the results to build theories and explanations (Popay et al. 1998). One study reported only a small number of examples of participant quotations to support the findings and was assessed as being moderately adequate (Cornsweet Barber et al. 2013).

## Synthesis of the findings

Data analysis revealed five descriptive themes: (1) motives and barriers to participating in studies, (2) acceptability of interventions, (3) satisfaction with components of interventions, (4) overall satisfaction with interventions and (5) perceived benefit from participation. Table 4 outlines the reported positive views of intervention components and highlights components which were less beneficial or acceptable. The CERQual assessment of the confidence in the evidence contributing to the findings is presented in Table 5.

**Table 4:** Summary of the themes and data from the included studies

First author country, year	Intervention description	Motives and barriers to participating in studies	Acceptability of interventions	Satisfaction with interventions	Perceived benefit from participation
*Interventions for women with elevated anxiety scores or risk factors*					
Goodman US 2014	Mind body Group mindful CBT		Women said the amount of home practice was sometimes too much. They suggested including partners in one session. Some would like ongoing support for their mindfulness practices.	Most women benefited from the experience. Would recommend to friends.	Some women said they learnt different options to deal with anxiety. They developed acceptance of their feelings and were kinder to themselves. Interaction within a supportive group reduced their feelings of isolation.
*Interventions for women with elevated anxiety and depression scores or risk factors*					
Bittner Germany 2014	Psychological Group CBT			Most women were satisfied with the intervention.	Most women found the intervention beneficial.
Milgrom Australia 2015	Psychological Individual CBT			Most women were satisfied with the intervention.	Most women found the intervention effective and helpful
Thomas Australia 2014	Psychological*/*Educational Group, Behavioural, IPT, psycho-educational.	Reasons for declining to participate included: work commitments, unsuitable timing of sessions, childcare issues, lack of interest or clash with other antenatal appointments		Most women were highly satisfied, and the intervention had met their expectations.	
Davis US 2015	Mind body Group yoga	Women attended an average of 6 out of 8 classes. Reasons for missed classes included travelling and illness.		Most participants found the intervention to be highly credible and were satisfied with the intervention.	
Woolhouse Australia 2014	Mind body Group mindfulness	The opportunity to learn new skills was a common motivation for participation. Women wanted to learn ways to manage mental health challenges.	Some exercises were challenging. Women engaged in different ways, picking the best exercises for them. Group participation was initially uncomfortable, but ultimately enjoyable.		Mindfulness (Body Scan) helped some women to sleep. They valued developing an ability to reflect on their emotions. Some reported improved relationships with family and colleagues. They felt able to respond to challenging situations.
Darwin 2013 UK	Other Self-report psychological assessment	Some women were concerned that disclosing their distress may lead to interference by social services or HCPs. Other women were concerned that their feelings would be dismissed	Some women valued interactions where HCPs listened rather than psychosocial assessment being viewed a routine. Some felt confronted by their distress following assessments without the offer of further support. Assessment was often completed without discussion.		The interview enabled some women to reflect about their thoughts and feelings. For some it was the first opportunity to talk about their feelings and experiences. Some women embraced self-reflection through the questionnaires
Breustedt Scotland, UK 2013	Psychological*/*Social Support Group Psychological, IPT, practical techniques	Some women were uncertain of the reason for referral to the intervention and felt pressured to attend. They feared judgement from other group participants.	Women described the groups created a relaxed, non-judgemental atmosphere. Home visits helped create a welcoming experience.		Some women described the groups as an accepting atmosphere to share experiences. They addressed issues difficult to discuss with others and reduced women’s feelings of isolation.
First author country, year	Intervention description	Motives and barriers to participating in studies	Acceptability of interventions	Satisfaction with interventions	Perceived benefit from participation
*Interventions for women with elevated depression scores or risk factors*					
McGregor Canada 2014	Psychological Individual CBT		Reasons for withdrawing included not having time to complete homework. Some women would have liked more time and in-depth discussions with their physician about their mood difficulties.		Some women said the intervention helped them be aware of their moods and subsequently were able to change their mood in a positive direction.
*Interventions for women with a history of pregnancy loss*					
Côté-Arsenault US 2014	Psychological Individual supportive interactions		Home visits, pregnancy diary, relaxation and problem solving exercises received positive comments. Women found visualisation exercises somewhat difficult. Fetal movement counting was reassuring although women felt anxious until they felt their baby move. Some valued learning assertiveness techniques.	Most women found participation easy and the home visits were described as valuable. Women in the control group were disappointed that they did not receive an intervention but grateful research was being done.	The women found the nurse non-judgmental, knowledgeable, and supportive. They reported reduced feelings of isolation, stress, anxiety and greater confidence. Women felt more positive about pregnancy and the intervention helped to normalise their anxiety. Completing the diary helped them reflect on their feelings over the pregnancy.
*Interventions for a general population of pregnant women*					
Brugha UK 2015	Psychological Enhanced psychological training of community midwives (assessment, CBA)	Some women had not felt the need to share their feelings but felt they had the support if needed. Where women felt they would not have been able to share their feelings, it was attributed to the fact that they had not built a relationship with the CMW.	One woman offered CBA commented that two home visit sessions were sufficient for her needs. Women mostly found the EPDS helpful and important. A few women did not find it easy to discuss their emotions.	Most women valued the CMW exploring and discussing their feelings and welcomed the availability of support. Women were mainly positive about CMWs administering the EPDS.	For home visits, women mostly felt that CMWs were open, caring and supportive. Home visits offered reassurance and guidance. The EPDS increased women’s awareness of their moods and anxiety. Women appreciated that support was available if required.
Beddoe US 2009	Mind body Group mindfulness and yoga		Women who lived further away found sessions difficult to attend.	Most participants were satisfied and would recommend the intervention to other women	Most women felt more hopeful and confident and said they were taking better care of themselves. They developed awareness about the sources of their stress which helped them to cope with stressful situations.
Cornsweet Barber New Zealand 2013	Mind body Individual mindfulness and relaxation		Initial frustration with completing exercises, but it became easier. Some said the language used was confusing. One participant did not complete all content.	All women found the intervention enjoyable, would recommend to others.	Women said the exercises were helpful to do before sleeping. Some felt the exercises might be helpful during labour.
Dunn Australia 2012	Mind body Group mindfulness	Women with a history of anxiety or depression had increased interest in and engagement with the intervention. Wanted to create a positive pregnancy experience.		Most women valued group participation and forming new relationships.	Sharing experiences and stories with the group had the benefit of normalising women’s own experience.

Green boxes display positive views on intervention components

Red boxes display intervention and areas which were less beneficial or acceptable

*HCP* healthcare professional, *CMW* community midwife, *CBA* cognitive-based approach, *CBT* cognitive-based therapy, *IPT* inter-personal therapy, *EPDS* Edinburgh postnatal depression scale (Cox et al. 1987)

**Table 5:** GRADE-CERQual assessment of the themes identified in the findings

Acceptability of and perceived benefit of interventions	Confidence in the evidence	Relevant papers	Explanation of confidence in the evidence assessment
Groups and individual home visits by HCPs provided an opportunity to discuss emotional issues which women found difficult to discuss with others. Discussions and supportive interactions reduced feelings of isolation.	High confidence	(Breustedt and Puckering 2013, Brugha et al. 2015, Côté-Arsenault et al. 2014, Dunn et al. 2012, Goodman et al. 2014, Woolhouse et al. 2014)	In general the studies were moderately well conducted. The finding was seen across most studies and settings.
Most women were satisfied with interventions which they found enjoyable and would recommend to others.	High confidence	(Brugha et al. 2015, Cornsweet Barber et al. 2013, Côté-Arsenault et al. 2014, Davis et al. 2015, Dunn et al. 2012, Goodman et al. 2014, Milgrom et al. 2015, Woolhouse et al. 2014)	In general the studies were moderately well conducted. The finding was seen across most studies and settings.
Initially women had concerns about disclosing their symptoms. They feared the judgement of others (in group interventions) and interference from HCPs.	Moderate confidence	(Breustedt and Puckering 2013, Darwin et al. 2013, Woolhouse et al. 2014)	In general the studies were moderately well conducted. The finding was seen across several studies and settings.
Mindfulness and CBT helped women to develop self-awareness and most women felt more positive and confident following the intervention.	Moderate confidence	(Breustedt and Puckering 2013, Côté-Arsenault et al. 2014, Goodman et al. 2014, Woolhouse et al. 2014)	In general the studies were moderately well conducted. The finding was seen across several studies and settings.
Women with history of anxiety/depression were motivated to participate in interventions.	Low confidence	(Dunn et al. 2012, Woolhouse et al. 2014)	In general the studies were moderately well conducted. The finding was seen across a few studies and settings.
Some CBT, mindfulness and relaxation exercises were initially challenging but became easier with practice.	Low confidence	(Cornsweet Barber et al. 2013, Woolhouse et al. 2014)	In general the studies were moderately well conducted. The finding was seen across a few studies and settings.
Women welcomed a choice of exercises and variety of techniques to practice.	Low confidence	(Goodman et al. 2014, Woolhouse et al. 2014)	In general the studies were moderately well conducted. The finding was seen across a few studies and settings.

### Motivation and barriers to participating in studies

Participants in studies of mindfulness interventions who had previous experience of anxiety and depression were motivated to participate (Dunn et al. 2012; Woolhouse et al. 2014). Women wanted to learn new ways to manage their symptoms; they considered that the intervention would help them achieve a positive experience of pregnancy. However, some women who were identified or referred for inclusion by a healthcare professional (HCP) had concerns about participation (Breustedt and Puckering 2013; Darwin et al. 2013). They were uncertain about the reason for their selection and were concerned that disclosing their symptoms may lead to unwanted interference from HCPs and social care services.

### Acceptability of interventions

Studies with reported attrition rates below 25% included group yoga interventions (Beddoe et al. 2009; Davis et al. 2015) and interventions provided one-to-one (Brugha et al. 2015; Cornsweet Barber et al. 2013; Côté-Arsenault et al. 2014; Milgrom et al. 2015). Five out of seven of the studies with lower attrition rates did not include psychological assessment as part of the inclusion criteria. Rates of attrition greater than 45% were reported in studies of a group CBT intervention for women with elevated anxiety and depression scores (Bittner et al. 2014) and a psycho-social intervention for women with complex social factors (Breustedt and Puckering 2013).

Women assessed as vulnerable or at risk of developing anxiety and depression initially felt uncomfortable attending group sessions and feared judgement or disapproval from the group (Breustedt and Puckering 2013; Woolhouse et al. 2014). Creating a relaxed and non-judgemental atmosphere and visiting the women at home before the group began helped women to feel confident about attending and created a welcoming experience. Once the group was established, sharing time with other pregnant women was valued by most participants (Breustedt and Puckering 2013; Dunn et al. 2012; Woolhouse et al. 2014).

### Satisfaction with components of interventions

Mcgregor et al. (2013) delivered a brief individual CBT intervention in 10-min sessions, but reported that some women would have liked more time and in-depth discussions about their emotional difficulties. Having time to discuss emotional issues with HCPs was highlighted as an important component by Darwin et al. (2013). Research interviews provided women with an opportunity to talk, which for some had been the first opportunity to discuss their feelings.

A number of participants in the study by Darwin et al. (2013) felt that completing psychological questionnaires resulted in them being confronted by the reality of their anxiety and depressive symptoms but they felt left without any further support. Brugha et al. (2015) reported that many women found completing the EPDS important and helpful. However, a few women found it difficult to discuss their emotions and felt apprehensive about the potential consequences resulting from elevated EPDS scores, such as the information being used by HCPs to raise child protection concerns.

Breustedt and Puckering (2013) discussed how the end of the group left some participants with a sense of loss and signalled a period of adjustment. This was addressed by the provision of follow-up postnatal groups and reunions. Some women in the study by Goodman et al. (2014) suggested that having partners included in at least one session would help support them with their new practices and would have welcomed on-going support to continue developing mindfulness techniques.

Some studies of mind-body interventions included homework exercises. Authors reported that participants had not completed some of the content (Cornsweet Barber et al. 2013) or at times, the homework had felt too much for the women to complete (Goodman et al. 2014). Certain exercises were reported as helpful to some women and unhelpful to others; however, women did not feel any specific exercises should be omitted. Women wanted an opportunity to learn a variety of techniques, having the choice to participate in exercises which they enjoyed or found useful (Goodman et al. 2014; Woolhouse et al. 2014).

### Overall satisfaction with interventions

Women who participated in psychological or mind-body interventions reported an overall satisfaction and described interventions as enjoyable, valuable and beneficial. Group interventions received positive comments, women were able to discuss their thoughts and experiences which they had found difficult to discuss with professionals or their family (Breustedt and Puckering 2013). Groups provided a supportive environment where they could make friends, knowing that others had similar thoughts and experiences helped women develop an acceptance of their feelings and feel less isolated (Breustedt and Puckering 2013; Dunn et al. 2012; Goodman et al. 2014).

### Perceived benefit from participation

Some women felt they had derived benefit from learning practical breathing techniques and developing an ability to reflect on their thoughts and emotions (Cornsweet Barber et al. 2013; Woolhouse et al. 2014). Women said that exercises such as the body scan (being aware of different areas of the body) had helped them to sleep better.

Some participants in the studies of mindfulness and CBT interventions reported a greater understanding of the causes of stress and anxiety in their lives and greater self-awareness of their thought patterns. This helped them respond in a more positive way to situations and feelings, before negative thought patterns could escalate (Beddoe et al. 2009; Goodman et al. 2014; McGregor et al. 2013; Woolhouse et al. 2014). For some women, learning to recognise their feelings helped them to accept their anxious thoughts (Goodman et al. 2014). Rather than becoming annoyed or frustrated, they had learned to be kinder to themselves and felt more confident and positive about the future (Beddoe et al. 2009; Breustedt and Puckering 2013; Côté-Arsenault et al. 2014).

## Discussion

The review was conducted to evaluate women’s views on the acceptability of and satisfaction with non-pharmacological interventions to reduce the symptoms of anxiety in pregnancy. Fourteen studies from six countries were included which accessed women’s views through qualitative interviews or questionnaires.

The review followed a narrative synthesis framework (Popay et al. 2006) and used the CERQual approach to assess the confidence in the findings of the review. Themes assessed as having a high confidence were seen in at least six of the included studies, all of which were assessed as being at least moderately well conducted.

### Quality of included studies

Only two survey studies used validated questionnaires to access participant feedback. Such feedback can be used to improve intervention design, recruitment of and study retention in clinical trials. However, validated surveys and benchmarks need to be developed to assess the experience of participation in clinical trials (Planner 2015). In many of the studies, data were collected from all or a sub-section of participants who had successfully completed interventions which was a potential source of selection bias. Five of the 14 studies collected data from all or a sub-set of participants in the postnatal period which may introduce positive or negative recall bias.

Recruitment and data collection methods were only described in three studies. Four of the seven studies which used qualitative interviews to access women’s views provided detailed descriptions of the analytic method. All of the qualitative studies presented participant quotations to support the findings. Lewin et al. (2009) described how qualitative components are included in RCTs of complex interventions to explore participants’ experiences; however, the quality of qualitative components can be variable and often lacks justification. Recent reviews of interventions focused on psychological health and wellbeing in pregnancy have highlighted the need to improve the reporting of study methods, recruitment strategies and study quality (Fontein-Kuipers et al. 2014; Marc et al. 2011; Morrell et al. 2016; Ryan 2013).

### Participants

Studies which included women from general antenatal populations aimed to help women develop coping strategies to prevent the development of symptoms of anxiety/depression, whereas, studies which recruited women with elevated scores or risk factors for anxiety and/or depression aimed to reduce or improve existing anxiety symptoms.

Milgrom et al. (2015) reported that 54% of the initial study population declined to complete symptom checklists; however, other studies which conducted psychological eligibility assessment did not report the rates of consent (Bittner et al. 2014; Goodman et al. 2014; McGregor et al. 2013). Reporting the rate for agreeing or declining eligibility assessment would help researchers to consider the design of effective recruitment strategies (Williams et al. 2007). Recruitment could be maximised through discussion and providing information early in the recruitment process, addressing women’s concerns about psychological screening and fear of stigma (Brintnall-Karabelas et al. 2012; NICE 2018). Women’s apprehensions about joining group interventions may be eased by conducting welcome visits, prior to group commencement, in order that women feel more confident to participate (Breustedt and Puckering 2013).

Only one study was focused on women with elevated symptoms of anxiety, with seven studies selecting women with symptoms or risk factors for anxiety alongside other psychosocial symptoms or risk factors. Although a multidimensional approach has been reported as an important factor to promote psychological wellbeing in pregnancy (Jomeen 2004), interventions targeting one condition may not be effective for the other co-morbid condition (Garber and Weersing 2010). Interventions that focus on improving symptoms of anxiety and depression need to define the underpinning theory of change before testing the mechanism by which an improvement in symptoms is likely to occur for each condition.

### Interventions

Only three studies reported details of the facilitator training to deliver interventions. In most studies, women were not asked to provide their views on the acceptability or relevance of intervention facilitators. Such information could be helpful for researchers to consider the type, skill requirement and appropriate expertise of intervention facilitators, making efficient use of the available resources.

Developing an awareness of the causes of anxiety and the ability to reflect on thoughts and emotions was reported as beneficial by women across all categories of interventions. Darwin et al. (2013) highlighted that some women felt distressed when confronted by their emotions and suggested that self-reflection needed to be followed with further support and discussion. Facilitating time for women to discuss their feelings and experiences was highlighted as an important component across the included studies. Discussions with HCPs were reported as helpful for women with symptoms of or risk factors for mental illness (Brugha et al. 2015; Côté-Arsenault et al. 2014; Darwin et al. 2013; McGregor et al. 2013). In group interventions, women who felt isolated found comfort when they discovered other women had similar thoughts and experiences (Breustedt and Puckering 2013; Dunn et al. 2012; Goodman et al. 2014; Woolhouse et al. 2014). Most studies of psychological and social support interventions included multiple components: psychological therapy, discussion sessions, parent education and/or social support. An investigation into the acceptability and satisfaction of specific components was only reported in the qualitative studies, possibly because these studies had greater scope to report in-depth qualitative findings.

The location of interventions and level of commitment were important factors for women (Beddoe et al. 2009; McGregor et al. 2013). Work commitments and other responsibilities may restrict women’s ability to regularly attend sessions and complete additional homework. Most interventions were held during the daytime in hospital clinics, although some were also offered in community centres and during the evening which may have made it easier for women to attend.

### Strengths of the review

To our knowledge, this is the only review of women’s views on the acceptability of and satisfaction with interventions to reduce the symptoms of anxiety in pregnancy. A comprehensive search strategy increased the likelihood that all potentially relevant studies were included. The review was strengthened by using good quality, independent and appropriate assessment methods. The use of the CERQual tool helped assess the certainty of the findings. A narrative synthesis approach (Popay et al. 2006) involved a textual and thematic exploration of the data, identifying common themes, contradictions and highlighting where the evidence was absent (Lucas et al. 2007). This helped to develop recommendations for the design and reporting of future research (Craig et al. 2008).

### Limitations of the review

Studies not published in English were not included in the review. Most of the included studies had small sample sizes (*n* = 4–30); many were feasibility studies or additional components to larger trials. Due to the limited reporting of the study methods in many of the included studies, a full quality assessment was not possible although methodological limitations were assessed and informed the overall CERQual findings. There was considerable heterogeneity between the intervention designs, participants and time frames in the included studies. Participation and experiences of interventions may differ for particular groups of women. The narrative synthesis explored and compared the different approaches to inform discussion and consideration of future intervention designs (Lucas et al. 2007).

## Conclusion

The review findings are limited due to the small number of included studies, many with small sample sizes and limited reporting of methods. Women’s views on the acceptability of and satisfaction with a range of interventions were overwhelmingly positive. The review has highlighted the importance of creating a welcoming non-judgemental context for group interventions. Most women valued individual or group discussions about their symptoms of anxiety. Discussions helped women to feel supported and develop supportive networks.

Responding to women’s views and experiences will help to inform the design of interventions which are acceptable to women and to develop an understanding of how and why intervention components may contribute to outcomes. Many qualitative studies accessed the views of women who had successfully completed interventions which introduced the potential for selection bias. Future studies need to access and report the views of women who did not participate or complete interventions to identify where further improvements are required. Researchers need to consider the acceptability of eligibility screening and identify ways to effectively communicate the purpose of screening to potential participants.

Study reports should include the methodological approach, recruitment strategy, intervention provider details and data analysis procedures. The use of validated evaluation questionnaires, following quality frameworks and reporting process evaluations will help researchers compare intervention studies and assess whether interventions may produce similar or different effects in other settings.

## Appendix 1

**Table 6:** Search strategy for MEDLINE

1	Intervention studies/or intervention*.mp
2	study.mp
3	clinical Trial/ or trial.mp
4	randomi*ed. controlled trial.mp
5	Randomised Controlled Trial as topic/ or rct.mp
6	review.mp
7	meta analysis.mp/or Meta-Analysis/
8	meta sysnthesis.mp
9	narrative synthesis.mp
10	systematic review.mp
11	Anxiety Disorders/ or anx*.mp or Anxiety/
12	qualitative.mp or Qualitative Research/
13	survey.mp
14	Patient Satisfaction/ or satisfaction*.mp
15	accept*.mp
16	perception*.mp
17	experience*.mp
18	attitude*.mp
19	view*.mp
20	Interview/ or interview*.mp
21	Focus Groups/ or focus group*.mp
22	findings.mp
23	pregnan*.mp
24	Pregnancy/ or childbearing.mp
25	Peripartum Period/ or peripart*.mp
26	perinatal.mp or Perinatal Care/
27	antenatal.mp or Prenatal Care/
28	ante-natal.mp
29	antepartum.mp
30	ante-partum.mp
31	1 or 2 or 3 or 4 or 5 or 6 or 7 or 8 or 9 or 10
32	12 or 13 or 14 or 15 or 16 or 17 or 18 or 19 or 20 or 21 or 22
33	23 or 24 or 25 or 26 or 27 or 28 or 29 or 30
34	11 and 31 and 32 and 33
35	Limit 34 to (English Language and female and humans and last 25 years)

## References

[CR1] American Psychiatric Association (2013). Diagnostic and Statistical Manual of Mental Disorders.

[CR2] Beck A, Epstein N, Brown G, Steer R (1988). An inventory for measuring clinical anxiety: psychometric properties. J Consult Clin Psychol.

[CR3] Beddoe A, Paul Yang C-P, Kennedy H, Weiss S, Lee K (2009). The effects of mindfulness-based yoga during pregnancy on maternal psychological and physical distress. J Obstet Gynecol Neonatal Nurs.

[CR4] Biaggi A, Conroy S, Pawlby S, Pariante C (2016). Identifying the women at risk of antenatal anxiety and depression: a systematic review. J Affect Disord.

[CR5] Bittner A, Peukert A, Zimmerman C, Junge-Hoffmeister C, Parker L, Stobel-Richter Y (2014). Early intervention in pregnant women with elevated anxiety and depressive symptoms. J Perinat Neonatal Nurs.

[CR6] Blair M, Glynn L, Sandman C, Davis E (2011). Prenatal maternal anxiety and early childhood temperament. Stress.

[CR7] Boynton Petra M, Greenhalgh Trisha (2004). Selecting, designing, and developing your questionnaire. BMJ.

[CR8] Breustedt S, Puckering C (2013). A qualitative evaluation of women’s experiences of the mellow bumps antenatal intervention. Br J Midwifery.

[CR9] Brintnall-Karabelas J, Sung S, Cadman M, Squires C, Whorton K, Pao M (2012). Improving recruitment in clinical trials: why eligible participants decline. J Empir Res Hum Res Ethics.

[CR10] Brugha T, Smith J, Austin J, Bankart J, Patterson M, Lovett C (2015). Can community midwives prevent antenatal depression? An external pilot study to test the feasibility of a cluster randomized controlled universal prevention trial. Psychol Med.

[CR11] Brunton RJ, Dryer R, Saliba A, Kohlhoff J (2015). Pregnancy anxiety: a systematic review of current scales. J Affect Disord.

[CR12] Cardwell M (2013). Stress: pregnancy considerations. Obstet Gynecol Surv.

[CR13] Centre for Reviews and Dissemination (2009). Systematic Reviews. CRD’s guidance for undertaking reviews in health care.

[CR14] Coelho H, Murray L, Royal-Lawson M, Cooper P (2011). Antenatal anxiety disorder as a predictor of postnatal depression: a longitudinal study. J Affect Disord.

[CR15] Cornsweet Barber C, Clark M, Williams S, Isler R (2013). Relaxation and mindfulness to manage computerised self-help programme. MIDIRS Midwifery Digest.

[CR16] Côté-Arsenault D, Krowchuk H, Schwartz K, McCoy T (2014). Evidence-based intervention with women pregnant after perinatal loss. Am J Matern Child Nurs.

[CR17] Cox J, Holden J, Sagovsky R (1987). Detection of postnatal depression. Development of the 10-item Edinburgh postnatal depression scale. Br J Psychiatry.

[CR18] Craske M, Rauch Ã, Ursano R, Prenoveau J, Pine D, Zinbarg R (2009). What is an anxiety disorder?. Depress Anxiety.

[CR19] Critical Appraisal Skills Programme (CASP) (2014) CASP Checklists. Oxford. UK

[CR20] Czarnocka J, Slade P (2000). Prevalence and predictors of post-traumatic stress symptoms following childbirth. Br J Clin Psychol.

[CR21] Darwin Z, McGowan L, Edozien L (2013). Assessment acting as intervention: findings from a study of perinatal psychosocial assessment. J Reprod Infant Psychol.

[CR22] Davis E, Sandman C (2010). The timing of prenatal exposure to maternal cortisol and psychosocial stress is associated with human infant cognitive development. Child Dev.

[CR23] Davis K, Goodman S, Leiferman J, Taylor M, Dimidjian S (2015). A randomized controlled trial of yoga for pregnant women with symptoms of depression and anxiety. Complement Ther Clin Pract.

[CR24] Department of Health (1999). National Service Framework: mental health.

[CR25] Department of Health (2009). Healthy child Programme: pregnancy and the first 5 years of life.

[CR26] Department of Health (2012). Maternal mental health pathway.

[CR27] Ding X-X, Wu Y-L, Xu S-J, Zhu R-P, Jia X-M, Zhang S-F, Huang K, Zhu P, Hao JH, Tao FB (2014). Maternal anxiety during pregnancy and adverse birth outcomes: a systematic review and meta-analysis of prospective cohort studies. J Affect Disord.

[CR28] Dunn C, Hanieh E, Roberts R, Powrie R (2012). Mindful pregnancy and childbirth: effects of a mindfulness-based intervention on women’s psychological distress and well-being in the perinatal period. Arch Womens Ment Health.

[CR29] Evans K, Morrell C, Spiby H (2015) Non-pharmacological interventions during pregnancy to reduce symptoms of anxiety: a systematic review of quantitative and qualitative evidence. Centre for Reviews aNd Dissemination*.* Prospero*.* Available at: http://www.crd.york.ac.uk/PROSPERO/display_record.asp?ID=CRD42015017841

[CR30] Fairbrother N, Janssen P, Antony MM, Tucker E, Young AH (2016). Perinatal anxiety disorder prevalence and incidence. J Affect Disord.

[CR31] Fontein-Kuipers Y, Nieuwenhuijze M, Ausems M, Budé L, de Vries R (2014). Antenatal interventions to reduce maternal distress: a systematic review and meta-analysis of randomised trials. BJOG.

[CR32] Furber C, Garrod D, Maloney E, Lovell K, McGowan L (2009). A qualitative study of mild to moderate psychological distress during pregnancy. Int J Nurs Stud.

[CR33] Garber J, Weersing VR (2010). Comorbidity of anxiety and depression in youth: implications for treatment and prevention. Clin Psychol Sci Pract.

[CR34] Glover V (2014). Maternal depression, anxiety and stress during pregnancy and child outcome; what needs to be done. Best Pract Res Clin Obstet Gynaecol.

[CR35] Goodman J, Chenausky K, Freeman M (2014). Anxiety disorders during pregnancy: a systematic review. J Clin Psychiatry.

[CR36] Heron J, O’Connor T, Evans J, Golding J, Glover V (2004). The course of anxiety and depression through pregnancy and the postpartum in a community sample. J Affect Disord.

[CR37] Highet N, Stevenson A, Purtell C, Coo S (2014). Qualitative insights into women’s personal experiences of perinatal depression and anxiety. Women Birth.

[CR38] Iles J, Slade P, Spiby H (2011). Posttraumatic stress symptoms and postpartum depression in couples after childbirth: the role of partner support and attachment. J Anxiety Disord.

[CR39] Jensen L, Allen M (1996). Meta-synthesis of qualitative findings. Qual Health Res.

[CR40] Jomeen J (2004). The importance of assessing psychological status during pregnancy, childbirth and the postnatal period as a multidimensional construct: a literature review. Clin Eff Nurs.

[CR41] Keeton C, Perry-Jenkins M, Sayer A (2008). Sense of control predicts depressive and anxious symptoms across the transition to parenthood. J Fam Psychol.

[CR42] Kroenke K, Spitzer R, Williams J (2001). The PHQ-9: validity of a brief depression severity measure. J Gen Intern Med.

[CR43] Lewin S, Glenton C, Oxman A (2009). Use of qualitative methods alongside randomised controlled trials of complex healthcare interventions: methodological study. BMJ.

[CR44] Lewin S, Glenton C, Munthe-Kaas H, Carlsen B, Colvin C, Gülmezoglu M, Rashidian A (2015). Using qualitative evidence in decision making for health and social interventions: an approach to assess confidence in findings from qualitative evidence syntheses (GRADE-CERQual). PLoS Med.

[CR45] Lewinsohn P, Antonuccio D, Steinmetz J,Teri L (1984) The coping with depression course: a psycho-educational intervention for unipolar depression. Castalsa Publishing Company, Eugene

[CR46] Littleton H, Breitkopf C, Berenson A (2007). Correlates of anxiety symptoms during pregnancy and association with perinatal outcomes: a meta-analysis. Am J Obstet Gynecol.

[CR47] Lucas P, Baird J, Arai L, Law C, Roberts H (2007). Worked examples of alternative methods for the synthesis of qualitative and quantitative research in systematic reviews. BMC Med Res Methodol.

[CR48] Marc I, Toureche N, Ernst E, Hodnett E, Blanchet C, Dodin S (2011). Mind-body interventions during pregnancy for preventing or treating women ‘s anxiety. Cochrane Database Syst Rev.

[CR49] Maternal Mental Health Alliance, NSPCC & Royal College of Midwives (2013) Specialist mental health midwives. Maternal mental health Alliance

[CR50] McGregor Marla, Coghlan Michelle, Dennis Cindy-Lee (2013). The effect of physician-based cognitive behavioural therapy among pregnant women with depressive symptomatology: a pilot quasi-experimental trial. Early Intervention in Psychiatry.

[CR51] Meyer T, Miller M, Metzger R, Borkovec T (1990). Development and validation of the Penn State worry questionnaire. Behav Res Ther.

[CR52] Milgrom J, Holt C, Holt C, Ross J, Ericksen J, Gemmill A (2015). Feasibility study and pilot randomised trial of an antenatal depression treatment with infant follow-up. Arch Womens Ment Health.

[CR53] Moher D, Liberati A, Tetzlaff J, Altman D (2009). Preferred reporting items for systematic reviews and meta-analyses: the PRISMA statement. PLoS Med.

[CR54] Morrell C, Warner R, Slade P, Dixon S, Walters S, Paley G (2009). Psychological interventions for postnatal depression: cluster randomised trial and economic evaluation. The PoNDER trial. Health Technol Assess.

[CR55] Morrell C, Sutcliffe P, Booth A, Stevens J, Scope A, Stevenson M, Stewart-Brown S (2016). A systematic review, evidence synthesis and meta-analysis of quantitative and qualitative studies evaluating the clinical effectiveness, the cost-effectiveness, safety and acceptability of interventions to prevent postnatal depression. Health Technol Assess.

[CR56] National Institute for Health and Care Excellence (2011). Common mental health problems : identification and pathways to care.

[CR57] National Institute for Health and Care Excellence (2018) Antenatal and postnatal mental health: clinical management and service guidance. NICE clinical guideline. NICE, London 31990493

[CR58] National Institute for Health and Clinical Excellence (2010). Pregnancy and complex social factors. NICE clinical guideline.

[CR59] Planner C (2015). Measuring patients’ experience of clinical trials: results of an exploratory review and stakeholder workshop. Trials.

[CR60] Popay J, Rogers A, Williams G (1998). Rationale and standards for the systematic review of qualitative literature in health services research. Qual Health Res.

[CR61] Popay J, Roberts H, Sowden A, Petticrew A, Arai L, Rodgers M, Duffy S (2006) Guidance on the conduct of narrative synthesis in sytematic reviews. Institute for Health Research, London

[CR62] Rich-Edwards J, Grizzard T (2005). Psychosocial stress and neuroendocrine mechanisms in preterm delivery. Am J Obstet Gynecol.

[CR63] Rubertsson C, Hellstrom J, Cross M, Sydsjo G (2014). Anxiety in early pregnancy: prevalence and contributing factors. Arch Womens Ment Health.

[CR64] Ryan A (2013) Interventions to reduce anxiety during pregnancy: an overview of research. Perspective - NCT’s journal on preparing parents for birth and early parenthood, June: 16–20

[CR65] Segal Z, Williams J, Teasdale J (2002). Preventing depression:mindfulness-based cognitive therapy.

[CR66] Spielberg C, Gorsuch R, Lushene R (1970). State trait anxiety inventory. Manual for the state trait anxiety inventory.

[CR67] Spitzer R, Kroenke K, Williams J, Löwe B (2006). A brief measure for assessing generalized anxiety disorder: the GAD-7. Arch Intern Med.

[CR68] Staneva A, Bogossian F, Wittkowski A (2015). The experience of psychological distress , depression , and anxiety during pregnancy: a meta-synthesis of qualitative research. Midwifery.

[CR69] Stein A, Pearson R, Goodman S, Rapa E, Rahman A, Mccallum M, Pariante C (2014). Perinatal mental health: eff ects of perinatal mental disorders on the fetus and child. Lancet.

[CR70] Swanson Kristen M. (1993). Nursing as Informed Caring for the Well-Being of Others. Image: the Journal of Nursing Scholarship.

[CR71] Higgins J, Green S, The Cochrane Collaboration (2011). Cochrane Handbook for Systematic Reviews of Interventions Version 5.1.0.

[CR72] Thomas N, Komiti A, Judd F (2014). Pilot early intervention antenatal group program for pregnant women with anxiety and depression. Arch Womens Ment Health.

[CR73] Vythilingum B (2009). Anxiety disorders in pregnancy and the postnatal period. Continuing Medical Education (CME).

[CR74] Walsh D, Downe S (2005). Meta-synthesis method for qualitative research : a literature review. J Adv Nurs.

[CR75] Williams B, Irvine L, McGinnis A, McMurdo M, Crombie I (2007). When “no” might not quite mean “no”; the importance of informed and meaningful non-consent: results from a survey of individuals refusing participation in a health-related research project. BMC Health Serv Res.

[CR76] Williams C, Cantwell R, Robertson K (2008). Overcoming postnatal depression: a five areas approach.

[CR77] Woolhouse H, Mercuri K, Judd F, Brown S (2014). Antenatal mindfulness intervention to reduce depression, anxiety and stress: a pilot randomised controlled trial of the MindBabyBody program in an Australian tertiary maternity hospital. BMC Pregnancy Childbirth.

[CR78] Yali A, Lobel M (1999). Coping and distress in pregnancy: an investigation of medically high risk women. J Psychosom Obstet Gynaecol.

